# The Hemoglobin Wayne Variant and Association With Falsely Elevated HbA_1c_

**DOI:** 10.1210/jcemcr/luad043

**Published:** 2023-05-08

**Authors:** Neha Mulpuri, Ananda Bryant, Daryoush Shahin, Kyaw Soe

**Affiliations:** UT Southwestern Medical Center, Department of Internal Medicine, Dallas, TX 75390, USA; VA North Texas Health Care System, Division of Endocrinology, Dallas, TX 75216, USA; VA North Texas Health Care System, Division of Endocrinology, Dallas, TX 75216, USA; UT Southwestern Medical Center, Department of Internal Medicine, Dallas, TX 75390, USA; VA North Texas Health Care System, Division of Endocrinology, Dallas, TX 75216, USA

**Keywords:** type 2 diabetes mellitus, hemoglobinopathies, hemoglobin A_1c_

## Abstract

The objective of this work is to explain the effect of the clinically silent hemoglobinopathy hemoglobin Wayne (Hb Wayne) variant on glycated hemoglobin A_1c_ (HbA_1c_) assay. This variant can result in falsely high HbA_1c_ values among euglycemic individuals without diabetes mellitus (DM). We discuss 3 patients who were diagnosed with type 2 DM based on spuriously high HbA_1c_ values due to the presence of Hb Wayne. All 3 patients were found to have elevated HbA_1c_ values that did not correlate with other glycemic parameters such as capillary blood sugar, 2-hour oral glucose tolerance test, and fructosamine levels. Hemoglobin electrophoresis revealed that each patient had a rare hemoglobinopathy called Hb Wayne variant. These patients were reassured that they did not have DM and were able to avoid unnecessary treatment. These cases emphasize the importance of clinical judgment in recognizing the limitations and caveats of the HbA_1c_ test. It is always necessary to investigate further any discordance between HbA_1c_ values and the clinical picture or other glycemic parameters.

## Introduction

Diabetes mellitus (DM), a disorder affecting more than 10% of the US population, continues to be a major cause of morbidity and mortality worldwide. Since its introduction as a biomarker for glycemic control in 1976, glycated hemoglobin A_1c_ (HbA_1c_) has been a useful method for the assessment of diabetes control and long-term complications. In 2010, the World Health Organization and the American Diabetes Association defined criteria for diagnosis of DM as an HbA_1c_ greater than 6.5%. However, several conditions such as anemia, splenomegaly, asplenia, chronic alcoholism, lead poisoning, blood transfusion, and hemoglobinopathies can interfere with the accuracy of HbA_1c_ assay methods. Hemoglobin Wayne (Hb Wayne) is a rare, silent, alpha chain variant caused by a frameshift mutation in the *HbA2* gene. Although clinically silent, this mutation may falsely elevate HbA_1c_ values. Other glycemic control indices such as continuous glucose monitoring data, capillary blood sugar values, and fructosamine levels can be used to verify the accuracy of HbA_1c_ assay methods.

## Case Presentation

### Case 1

A 76-year-old White man with a history of coronary artery disease (CAD) was referred to the diabetes clinic for newly diagnosed type 2 DM (T2DM). His HbA_1c_ results were elevated to 9.9% to 10.1% (84.7-86.9 mmol/mol). He had a normal HbA_1c_ of 5.2% (33.3 mmol/mol) in 2004, but was not screened for diabetes regularly until he started seeing a primary care physician recently. The patient was lean and had no features of insulin resistance nor any family history of T2DM. He was not experiencing any signs or symptoms suggestive of T2DM. Review of the laboratory workup showed that renal function and complete blood count were within the normal range. Fasting plasma glucose was 82 to 94 mg/dL (4.55-5.22 mmol/L). Random glucose after breakfast was 89 mg/dL (4.94 mmol/L) the day of the visit. Two-hour glucose tolerance test (90 mg/dL [5 mmol/L] fasting and 99 mg/dL [5.49 mmol/L] 2-hour) and fructosamine level (263 μmol/L) were normal. Based on the discrepancy in HbA_1c_, fasting glucose, and lack of symptoms, the possibility of a confounding factor such as hemoglobinopathy was investigated. The repeat HbA_1c_ with a different assay (enzymatic assay) at Quest diagnostic laboratory also came back normal at 5.4% (35.5 mmol/mol). Hemoglobin electrophoresis confirmed the presence of Hb Wayne I variant (alpha Hgb variant 7.7%). Subsequently, the patient was informed that his HbA_1c_ was falsely elevated due to silent hemoglobinopathy. The patient was reassured and educated about his Hb variant, and no further treatment for T2DM was pursued.

### Case 2

A 70-year-old White man (White and Native American ethnicity) with a history of subclinical hyperthyroidism and CAD was diagnosed with diabetes 1 year ago. The original treatment plan included dietary and lifestyle modifications. Given persistently high HbA_1c_ values ranging from 10.3% to 10.6% (89.1 mmol/mol to 92.4 mmol/mol), he was then started on metformin 1000 mg twice per day and referred to the diabetes clinic. He checked his finger-stick blood glucose (FSBG) 1 or 2 times daily, and his glucose log showed normal fasting capillary blood sugar readings between 83 and 100 mg/dL (4.61-5.55 mmol/L) and normal random capillary blood sugar readings between 100 and 110 mg/dL (5.55-6.11 mmol/L). The patient was also noted to have normal renal function and normal complete blood count. Because of the discrepancy between HbA_1c_ and FSBG values, further workup was completed. Both fructosamine level (235 μmol/L) and HbA_1c_ from Quest diagnostic laboratory (5.5% or 36.6 mmol/mol) were normal. Hb electrophoresis revealed an abnormal alpha variant consistent with Hb Wayne. The patient was reassured about the situation and educated about this silent variant of Hb.

### Case 3

A 66-year-old White man with a history of CAD and hyperlipidemia was referred to the diabetes clinic for discrepancy between high HbA_1c_ values (10.4%-10.6% or 90.2-92.4 mmol/mol) and normal fasting plasma glucose readings between 74 and 96 mg/dL (4.11-5.33 mmol/L). The patient was very lean, muscular, and had no features of insulin resistance. The only family member with diabetes was his older brother, who was 60 pounds (27 kg) heavier than the patient. Further workup showed normal 2 hour oral glucose tolerance test (99 mg/dL [5.49 mmol/L] fasting and 107 mg/dL [5.49 mmol/L] at 2 hours after 75-g oral glucose intake). Fructosamine level (214 μmol/L) and repeat HbA_1c_ at Quest diagnostic laboratory (5.5% or 36.6 mmol/mol) were normal. Hb electrophoresis pattern was consistent with Hb Wayne. This confirmed that the patient did not have diabetes, and his primary care physician was recommended to use a different HbA_1c_ assay method from Quest for future laboratory testing.

## Discussion

HbA_1c_ is the most abundant minor component of Hb in human red blood cells, accounting for about 5% of the total Hb [1]. This type of Hb is elevated in DM due to nonenzymatic glycation of the terminal amino group of the beta chain. The higher HbA_1c_ reflects elevated plasma glucose levels over a 120-day period (the lifetime of a red blood cell). HbA_1c_ has been used for the diagnosis and assessment of glycemic control. The widely used methods for measurement of HbA_1c_ include immunoassay, ion-exchange high-performance liquid chromatography (HPLC), boronate affinity HPLC, and enzymatic assays [2]. Any alteration in the structure of Hb can affect the glycosylation process or red blood cell turnover, thus affecting HbA_1c_ levels and leading to incorrect diagnosis and treatment of diabetes. Hemoglobinopathies can result in spuriously high or low HbA_1c_ values depending on variants of hemoglobinopathy and type of HbA_1c_ assay. It is well known that HbA_1c_ value can be significantly underestimated among patients with Hb S or Hb C variants, the most common hemoglobinopathies. This is due to a decrease in HbA_1c_ when measured by cation-exchange (CE) chromatographic methods [3]. There are now resources available for clinicians to determine whether a method used to measure HbA_1c_ is affected by common hemoglobinopathies (Hb C, Hb S, Hb E, Hb D) through the National Glycohemoglobin Standardization Program (NGSP). However, a substantial number of rare hemoglobinopathies are clinically silent and can be missed easily.

**Figure 1.**
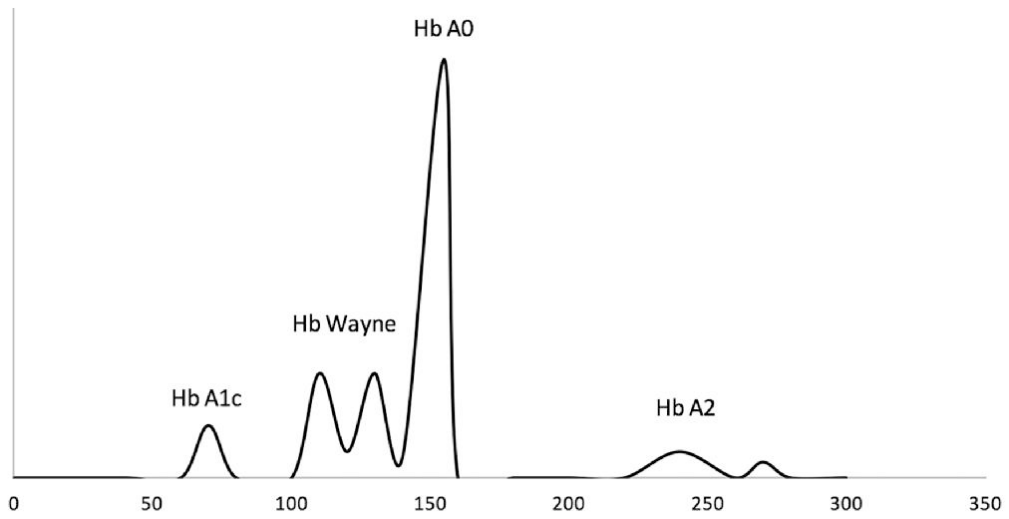
Hemoglobin electrophoresis of hemoglobin Wayne.

Hb Wayne is an alpha chain variant first described in 1976 [4]. It is due to a silent frameshift mutation in the *HbA2* gene (HbA1:c.420del). Due to the deamidation at asparagine 139, the Hb Wayne consists of 2 different isoforms known as Hb Wayne I (Asn 139) and Hb Wayne II (ASP 139) [5]. The Hb electrophoresis of Hb Wayne is shown in Fig. 1. While the prevalence of the Hb Wayne variant is unknown, at least 62 cases were reported over a 16-year period in the US population by analyzing almost 300 000 samples with the Bio-Rad Variant Classic HPLC instrument at Rochester Mayo Clinic laboratory [5]. Heterozygote carriers of this mutation are clinically normal and have no hematologic abnormalities. It is possible that a significant number of patients with specific silent Hb variants might be overdiagnosed with DM, especially within a certain ethnic group or in a particular geographic location. With regard to characteristics among patients with Hb Wayne in the literature, there is no specific demographic. Common characteristics include no symptoms related to DM, generally normal renal function/complete blood count, and in certain cases hypoglycemic episodes due to treatment of presumed DM. The 3 cases described in this paper were all diagnosed within the last 5 years at our diabetes clinic, and they highlight how this clinically silent variant can be encountered in daily clinical practice. All of the HbA_1c_ determination methods described in the aforementioned cases were NGSP certified.

While HbA_1c_ has been an important clinical tool for diagnosing and managing diabetes, clinicians should look for reasons that can affect the HbA_1c_ assay if there is any discrepancy between HbA_1c_ and measurements of glycemia, including capillary blood glucose, plasma glucose, or continuous glucose monitoring. Continuous glucose monitoring can also provide additional information to correlate with HbA_1c_. Additional tests such as Hb electrophoresis, DNA sequencing, and HPLC to specify any Hb variant should be pursued. In general, Hb variants should be suspected in extreme HbA_1c_ values greater than 15% or less than 4%. Each isoform of Hb Wayne comprises approximately 6% to 9% of total Hb, and only Hb Wayne I coelutes with HbA_1c_. Hence the spurious HbA_1c_ values are sometimes not extremely high and may not attract the attention of the laboratory technician to review manually. All 3 of our patients and previously published cases showed a similar HbA_1c_ range of 9% to 11% (74.9-96.7 mmol/mol).

Ce-HPLC of a red blood cell lysate is widely used in many laboratories because it is a rapid and reliable diagnostic technique to separate various Hb fractions. However, this method is susceptible to interferences by net charges due to amino acid substitution in hemoglobinopathies, resulting in an additional peak in the chromatogram. Amino acid substitution in Hb Wayne coelutes with HbA_1c_, resulting in similar retention time in platform and overestimation of HbA1c values. A double *HbA2* peak and the percentage of the major Hb variant can be helpful to recognize the presence of an alpha chain variant [5]. Interestingly, a recent case report suggests that the type of machine being used for HPLC may also change how the HbA_1c_ is affected by the presence of Hb Wayne [6]. For example, older HPLC instruments may be able to separate the Hb Wayne I protein peak from the normal Hb peaks, but the fast elution times of the newer HPLC instruments may result in overlap between Hb Wayne I with HbA_1c_ and mask the presence of an Hb variant. Additionally, compared to Ce-HPLC, boronate affinity chromatography may be less susceptible to interference by Hb variants.

Alternative methods of HbA_1c_ testing such as enzymatic assay, immunoassay, boronate affinity chromatography and capillary electrophoresis should be used for patients with Hb Wayne as they did not rely on net charges of the component of hemoglobin [7, 8]. In our cases, the laboratory at our institution uses the Ce-HPLC method (Bio-Rad D-100 instrument) resulting in falsely high HbA_1c_ values. The repeat HbA_1c_ test at Quest Diagnostics using an enzymatic assay (Architect 4000 instrument) was not affected by the Hb Wayne variant.

More than 1000 Hb variants have already been discovered, and a considerable number of them are clinically silent [9]. It is also worth noting that clinically silent Hb variants such as Hb Graz, Hb Sherwood Forest, Hb D, and Hb O Padova migrate like HbA_1c_ on chromatogram and can also result in inaccurate HbA_1c_ values depending on the assay method [10].

Falsely elevated HbA_1c_ can lead to unnecessary treatment that in turn has the potential to result in life-threatening complications such as hypoglycemia and coma. Hb Wayne can confound the HbA_1c_ measurement through some measurement techniques. Therefore, whenever there is discordance between HbA_1c_ and other glycemic indices among asymptomatic patients, silent hemoglobinopathies including the Hb Wayne variant should be considered. After diagnosis of such a rare Hb variant, it is also important to educate patients and their health care providers about the interference of their silent Hb variant on their specific HbA_1c_ assay. Health care providers should also be informed to use the specific HbA_1c_ assay unaffected by Hb Wayne. These 3 cases emphasize the importance of clinicians using clinical acumen to interpret laboratory results and find a meaningful correlation between laboratory values and overall clinical picture.

## Learning Points

- Rare hemoglobinopathies may be more common than previously expected as they can be clinically silent and easily overlooked.
- Some hemoglobinopathies can affect widely used HbA_1c_ testing methods such as Ce-HPLC. The effect on HbA_1c_ values will differ depending on the Hb variant and specific method and assay used.
- Given the limitations of HbA_1c_ testing, clinicians should avoid relying heavily on HbA_1c_ values in the management and diagnosis of diabetes. It is vital that clinicians use their clinical judgment to correlate HbA_1c_ with other clinical parameters such as continuous glucose monitoring data, glycated albumin, 1,5 anhydroglucitol (1,5 –AG), FSBG, plasma glucose, and fructosamine values.
- Clinicians can use widely available HbA_1c_ testing methods such as Ce-HPLC, though there should be a low threshold for repeating the test with different assay and methods such as enzymatic assay if there is discordance between clinical presentation and laboratory values.

## Contributors

All authors made individual contributions to authorship. N.M., K.S., A.B., and D.S. were involved in the writing of this manuscript. All authors reviewed and approved the final draft.

## Data Availability

Data sharing is not applicable to this article as no data sets were generated or analyzed during the current study.

## Abbreviations

CAD: coronary artery disease
Ce-HPLC: cation-exchange high-performance liquid chromatography
DM: diabetes mellitus
FSBG: finger-stick blood glucose
HbA_1c_: glycated hemoglobin A_1c_
Hb Wayne: hemoglobin Wayne
HPLC: high-performance liquid chromatography
NGSP: National Glycohemoglobin Standardization Program
T2DM: type 2 diabetes mellitus

## References

[1] Milhem M, July M, Elhamdani S, BenHamed N. Hemoglobin Wayne variant interfering with hemoglobin A1c measurement. AACE Clin Case Rep. 2019;5(1):e59‐e61. 31967002 10.4158/ACCR-2018-0017 PMC6876977

[2] Little RR, Roberts WL. A review of variant hemoglobins interfering with hemoglobin A1c measurement. J Diabetes Sci Technol. 2009;3(3):446‐451. 20144281 10.1177/193229680900300307 PMC2769887

[3] Aleyassine H. Low proportions of glycosylated hemoglobin associated with hemoglobin S and hemoglobin C. Clin Chem. 1979;25(8):1484‐1486. 455690

[4] Seid-Akhavan M, Winter WP, Abramson RK, Rucknagel DL. Hemoglobin Wayne: a frameshift mutation detected in human hemoglobin alpha chains. Proc Natl Acad Sci U S A. 1976;73(3):882‐886. 1062801 10.1073/pnas.73.3.882 PMC336023

[5] Szuberski J, Oliveira JL, Hoyer JD. A comprehensive analysis of hemoglobin variants by high-performance liquid chromatography (HPLC). Int J Lab Hematol. 2012;34(6):594‐604. 22713122 10.1111/j.1751-553X.2012.01440.x

[6] Chessler SD, Lee DE. Alarming increase in HbA1c and near misdiagnosis of diabetes mellitus resulting from a clinical laboratory instrument upgrade and haemoglobin variant. BMJ Case Rep. 2018;2018:bcr2018225358. 10.1136/bcr-2018-225358 PMC6011579 29903779

[7] Sharma P, Das R. Cation-exchange high-performance liquid chromatography for variant hemoglobins and HbF/A2: what must hematopathologists know about methodology? World J Methodol. 2016;6(1):20‐24. 27019794 10.5662/wjm.v6.i1.20 PMC4804249

[8] Strickland SW, Campbell ST, Little RR, Bruns DE, Bazydlo LAL. Recognition of rare hemoglobin variants by hemoglobin A_1c_ measurement procedures. Clin Chim Acta. 2018;476:67‐74. 29154790 10.1016/j.cca.2017.11.012

[9] Thom CS, Dickson CF, Gell DA, Weiss MJ. Hemoglobin variants: biochemical properties and clinical correlates. Cold Spring Harb Perspect Med. 2013;3(3):a011858. 10.1101/cshperspect.a011858 PMC3579210 23388674

[10] Schnedl WJ, Lahousen T, Wallner SJ, Krause R, Lipp RW. Silent hemoglobin variants and determination of HbA(1c) with the high-resolution program of the HPLC HA-8160 hemoglobin analyzer. Clin Biochem. 2005;38(1):88‐91. 15607323 10.1016/j.clinbiochem.2004.09.016

